# Chlorpromazine Sensitizes Progestin-Resistant Endometrial Cancer Cells to MPA by Upregulating PRB

**DOI:** 10.3389/fonc.2021.665832

**Published:** 2021-04-16

**Authors:** Yunxia Cui, Huiwen Wu, Linlin Yang, Ting Huang, Jian Li, Xiaodi Gong, Lijuan Li, Xiao Sun, Fei Mao, Yudong Wang

**Affiliations:** ^1^ Department of Gynecologic Oncology, The International Peace Maternity and Child Health Hospital, School of Medicine, Shanghai Jiao Tong University, Shanghai, China; ^2^ State Key Laboratory of Bioreactor Engineering, Shanghai Key Laboratory of New Drug Design, School of Pharmacy, East China University of Science and Technology, Shanghai, China; ^3^ College of Pharmacy and Chemistry, Dali University, Dali, China; ^4^ Frontiers Science Center for Materiobiology and Dynamic Chemistry, East China University of Science and Technology, Shanghai, China; ^5^ Shanghai Municipal Key Clinical Specialty, Female Tumor Reproductive Specialty, Shanghai, China

**Keywords:** endometrial cancer, chlorpromazine, medroxyprogesterone acetate, anticancer activity, sequential treatment

## Abstract

Medroxyprogesterone acetate (MPA) is the main conservative treatment for endometrial cancer (EC) patients desirable to preserve fertility and those who cannot suffer from surgery. Considering the high incidence of progestin resistance and recurrence of MPA treatment, we reproposed antipsychotics chlorpromazine (CPZ) as a new strategy for both progestin-sensitive and -resistant endometrial cancer. Cytobiology experiments indicated that CPZ could significantly suppress proliferation, migration/invasion and induce apoptosis in Ishikawa (ISK) and KLE EC cell lines. And xenograft mouse models were constructed to validate the antitumor effect and toxicity of CPZ *in-vivo*. CPZ inhibited the growth at a low dose of 3mg/kg and the mice exhibited no signs of toxicity. Next, concomitant treatment and sequential treatment with CPZ and MPA were proceeded to analysis the synergistic effect in EC cells. Concomitant treatment only performed a limited synergistic effect on apoptosis in ISK and KLE cells. Nevertheless, sequential treatment showed favorable synergistic effects in progestin-resistant KLE cells. Finally, a stable MPA-resistant cell line shRNA was established to explore the mechanism of CPZ reversing progestin resistance. Immunoblot data showed that CPZ inhibited the activation of PI3K/AKT signal in ISK and KLE cells and upregulated PRB expression in progestin-resistant cells, by which CPZ overcame progestin resistance to MPA. Thus, CPZ might act as a candidate drug for conservative treatment and sequential treatment with CPZ and MPA could be a suitable therapeutic option for progestin resistant patients.

## Introduction

Endometrial cancer (EC) is the most common gynecologic cancer in the United States (1); the incidence and mortality rates of this disease have increased gradually over the last decade (2). Although it typically occurs in postmenopausal women (3), EC is increasingly diagnosed in younger women (4, 5). Approximately 6.4% of all women with EC are diagnosed between 20 and 44 years old (6). With the implementation of the two-child policy, fertility preservation is more imperative and important than ever for young women in China. On the other hand, conservative treatment is in increasingly demand for patients who have lower operative-tolerance and those with advanced or recurring endometrial cancer.

Hormone therapy is the main regimen for these patients, the most common forms of which are medroxyprogesterone acetate (MPA) and levonorgestrel-releasing intrauterine devices (4, 7). MPA mainly exert anti-EC effects through progesterone receptor B (PRB). One major difference between type I and type II EC is the expression of PRB (hormone-receptor-positive and hormone-receptor-negative). For type I EC, although 70% of early patients respond to treatment, 57% of these patients experience recurrence. For advanced cases, the response rate is only 20–40% (8). Type II EC patients have a poor efficacy due to low expression of PRB and the response rate to hormone therapy was only 8% (9). Therefore, it is necessary to explore new strategies for cases of *de novo* or acquired progestin resistance.

Drug repurposing speeds up the overall development time and minimizes research expenses. Antipsychotic drugs such as olanzapine, risperidone and sertraline suppress tumor progress in several malignant disorders (10–12), which are expected to be a prospective reproposed antitumor drug. Unfortunately, there is no relevant study of such drugs in endometrial cancer. Our data showed that antipsychotic agents including trifluoperazine, thioridazine and perphenazine displayed anticancer activity in endometrial cancer (13, 14). Thioridazine could enhance the anticancer effect of MPA on the proliferative inhibition and apoptosis induction in ISK (type I) and KLE (type II) cells, which implied that thioridazine may act as a candidate drug for EC patients particularly the progestin-resistant ones. However, these drugs are rarely used in clinic due to the strong central nervous system side-effects (15–17). Therefore, reducing side effects (by shortening the duration or reducing the dosage) or developing new drugs with the same pesticide effect is an urgent need at the moment.

Considering that the above-mentioned three drugs with similar structure all belong to tricyclic antidepressants, we screened 20 tricyclic antipsychotic drugs in our approved drug library to discover a better candidate drug (**Supplementary Table 1**) (14). Chlorpromazine (CPZ) was found to exhibit better inhibitory activity. Given that a lower dosage of CPZ could play an effective anti-EC effect, CPZ was expected to decrease toxic side effects. Furthermore, CPZ could suppressed various cancers including glioblastoma, lung cancer, colon cancer and breast cancer (18–20). Therefore, we evaluated the possibility of reproposing CPZ in both progestin sensitive and resistant endometrial cancer in the present study.

## Materials and Methods

### Mice

We acquired female BALB/c nude mice from a commercial supplier (JieSiJie, Shanghai China). The mice needed to be at least 7 weeks-of age before they were included in experiments. For *in vivo* administration, ISK cells (that were >90% viable) were resuspended at a concentration of 1×10^8^ cells/ml in sterile phosphate buffered saline (PBS; pH 7.4). When tumor volumes were 50-150 mm^3^, the mice were randomly selected for a daily intraperitoneal injection of Mock (150 μL, n = 5 tumors), MPA (12mg/kg, n = 3 tumors), CPZ (3mg/kg, n = 5 tumors) or CPZ (12mg/kg, n = 5 tumors). Every three days, we measured the length (L) and width (W) of all tumors using external calipers. We also calculated the volume of all tumors by applying the following formula: tumor volume = L× W^2^× 0.5 (21), where length (L) is defined as the larger of the two measurements, and width (W) is defined as the smaller of the two measurements. After 21 days, all mice were sacrificed. Tissues were then gently resected and placed in paraformaldehyde for fixation. The animal experiments were approved by the Committee on the Ethics of Animal Experiments of Shanghai Jiao Tong University, China (GKLW 2018-40).

### Cell Cultures and Transfection

Human endometrial (ISK, KLE, HEC-1-A, HEC-1-B and AN3CA) cancer cell lines were obtained from the American Type Culture Collection (ATCC) and cultured in DMED-F12 medium (Hyclone, Logan, Calif) containing 100 U/mL penicillin, 10% fetal bovine serum (FBS), and 100μg/mL streptomycin, in a humidified incubator at 37°C with 5% CO_2_. The incubator was tested regularly for mycoplasma contamination. In order to establish a stable MPA-resistant cell line shRNA, pLKD-CMV-EFGP-2A-Puro-U6-PRB shRNA (OBiO Technology, Shanghai, China) was transfected into 293T cells (ATCC, Manassas, VA, USA) by using Opti-MEM (Gibco) and lipofectamine 2000 (Invitrogen). The infective virus was collected after transfection for 48 h. The ISK cells were infected at about 50% of the confluence. The virus solution was added with 5.0 μg/ml Polybrene (Sigma), and then screened with 1.0 μg/ml of puromycin (Sigma) for one week. The selected sequences for knockdown as follow: GCTGTAAGGTCTTCTTTAA.

### Chemicals and Antibodies

CPZ and MPA were purchased from Selleck (Selleck Chemicals, Shanghai, China). A range of antibodies were purchased from Zen BioScience (Chengdu, China) and Abcam (United Kingdom), including anti-PI3K (R22768), anti-AKT (R23412), anti-p-PI3K (341468), anti-p-AKT (381555), anti-PRB (ab2765), anti-Ki-67 (ab16667), anti-E-cadherin (ab76055) and anti-MMP-9 (ab228402). In Situ Cell Death Detection Kit, POD (11684817910) was purchased from Roche (Basel, Switzerland).

### CCK-8

Cells were aliquoted into 96-well plates at a concentration of 7×10^3^ cell/well. Cells then underwent their allocated experimental treatments. Following treatment, 10 μl of CCK-8 solution (Selleck Chemicals) was added to each well and incubated for 1 h at 37°C. Next, we measured the absorbance with a microplate reader at 450 nm.

### Transwell Assays

The upper chambers of a transwell insert (Corning, New York, NY, USA) were first seeded with cells and exposed to the indicated drugs for a 24-hour period; 600 μL of DMEM/F12 medium containing 10% FBS was added to the lower chambers. Following incubation, we fixed cells that had undergone migration in 4% paraformaldehyde and stained the fixed cells with 1% crystal violet. After staining, the cells were rinsed three times in PBS. An inverted microscope was then used to count the number of cells that had migrated. Invasion assays were carried out by applying 50 μl of Matrigel (BD, USA) to the upper chambers of the transwell inserts and then seeding with cells that had been starved. These cells were then treated with designated drugs for a 48-hour period; 600 μL of DMEM/F12 medium containing 20% FBS was added to the lower chambers. After incubation, we used cotton swabs to remove the Matrigel and all non-invading cells from the top of the polycarbonate membrane. We then carried out fixation and staining in the same manner as that described for the migration assays. An inverted microscope was used to acquire images that were representative of invasion.

### Flow Cytometry

For flow cytometry, we incubated cells with different concentration of CPZ and MPA for a total period of 72 h. Cells were then treated with pancreatin and centrifuged for collection. Harvested cells were then rinsed twice in PBS and stained for 15 min with Annexin V-FITC (BD Biosciences, San Diego, CA) and PI (BD Biosciences) in accordance with the manufacturer’s protocols. Stained cells were then quantified by flow cytometry.

### Clonal Formation Assays

Cells were seeded into 6-well plates (1000 cells/well) and cultured for indicated hours before being treated with the designated drugs. Cells were then cultured for another 7-10 days in a medium that was devoid of drugs until clones were formed (approximately 50 cells/clone). At this point, we used PBS to wash the cells which were finally fixed and stained with 5% crystal violet solution.

### Western Blot Analysis

After treatments with different agents, the cells were washed twice with PBS buffer. Total protein was extracted from cells with RIPA lysis buffer (containing phosphatase inhibitor and 1 μM PMSF) and then separated by SDS-PAGE. Separated proteins were then transferred electrophoretically onto polyvinylidene fluoride (PVDF) membrane. These membranes were then blocked for 2 h at room temperature and then incubated overnight at 4°C with primary antibodies. The following morning, membranes were incubated with appropriate second antibodies for a period of 2 h. Immuno-positive bands were then detected by ECL Western Blot kit (Leica, Germany).

### Immunohistochemistry

Samples of tumor tissue were fixed in 10% buffered formalin for a total period of 48h and then embedded in paraffin, sectioned into 5 μm slices, and then transferred to silane-coated slides. Sections were then deparaffinized and dehydrated before undergoing antigen retrieval at 95°C for 30 min. Following antigen retrieval, we used normal goat serum to block non-specific binding sites for one hour. Sections were then were incubated overnight at 4°C with primary antibodies and then incubated for 45 min at 37°C with HRP- conjugated secondary antibodies. Immuno-positive signals were then developed using DAB (Jiehao Biotechnology, China).

### HE Staining

Tissue sections (prepared from the tumor mass, lungs, spleen, kidneys, heart, and liver) were first hydrated and then stained in Mayer’s hematoxylin for 30 s with gentle agitation. Slides were then rinsed with water for 1 min and then stained with 1% eosin Y solution for 10−30 s with gentle agitation. Next, the sections were dehydrated by a double exposure to 95% alcohol for 30 s followed by a double exposure to 100% alcohol for 30 s. The alcohol was then removed, and the sections treated twice with xylene. Finally, tissue sections were mounted in an appropriate medium and cover-slipped.

### Biochemical and Hematological Analyses

Samples of whole blood were collected from all mice by cardiac puncture and aliquoted into microfuge tubes and K2E microtainer tubes (BD, USA). The blood samples stored in microtainer tubes were used to evaluate a range of hematological parameters; these analyses involved a BC-2800vet Hematology Analyzer (Mindray, China). The blood samples retained in microfuge tubes were first centrifuged at 10,000 rpm for 10 min to prepare serum samples. These serum samples were then evaluated using a range of assay kits acquired from Rayto (China) which allowed us to determine the serum levels of aspartate aminotransferase (S03040), alanine aminotransferase (S03030), carbamide (S03036) and creatinine (S03076). All assays were carried out in accordance with the manufacturer’s protocols. Optical density was determined by a Chemray 800 Automatic Biochemistry Analyzer (Rayto, China). OD readings were then used to determine a range of biochemical indicators.

### Statistical Analysis

All statistical analyses were carried out using GraphPad Prism version 7.0 (La Jolla, California). Significant differences were then determined by either the Student’s t-tests, one-way analysis of variance (ANOVA) for single variables, two-way ANOVA for two variables; Tukey’s correction was used for multiple comparisons.

## Results

### CPZ Inhibited EC Cell Growth, Migration, and Invasion

**Figure 1:**
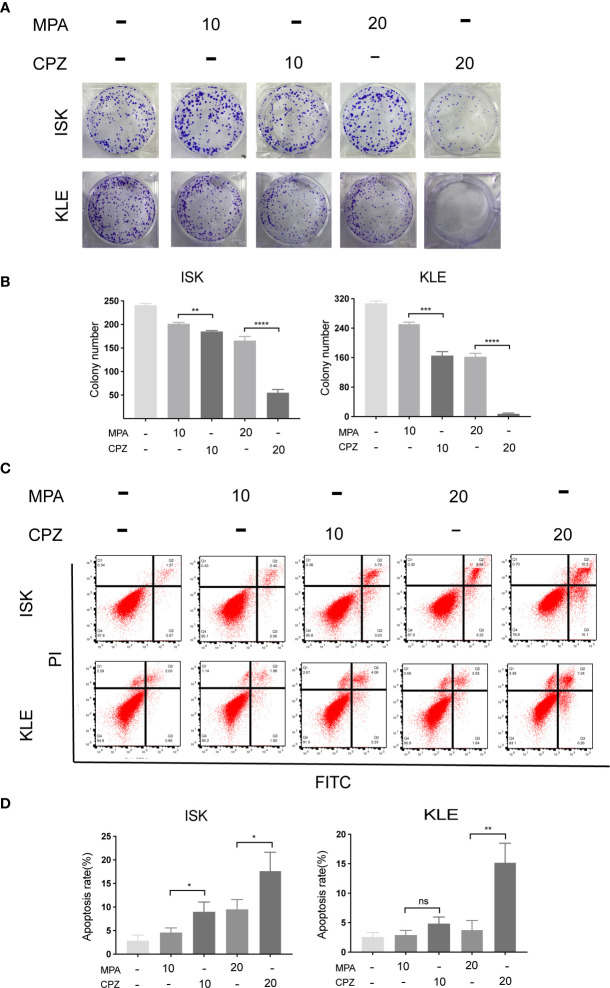
CPZ inhibits proliferation and promotes apoptosis of EC cells. **(A, B)** Cells were incubated with MPA (10, 20 μM) or CPZ (10,20μM) alone for 72 h. Colony formation assays were then utilized to evaluate the colony formation ability of EC cells in the presence of CPZ and MPA. **(C, D)** The rate of apoptosis was determined by flow cytometry. ISK and KLE cells were incubated with MPA (10, 20 μM) and CPZ (10,20μM) for a total of 72 h. Q1 represents necrotic cells, Q2 represents cells in the late phases of apoptosis, Q3 represents cells in the early phases of apoptosis, and Q4 represents normal cells. Columns ± bars: IC_50_ mean ± standard deviation. ns, not significant; ^*^p < 0.05; ^**^ p< 0.01; ^***^p < 0.001; ^****^p < 0.0001.

In order to compare the effect of MPA and CPZ on the EC cell proliferation, we treated different EC cell lines with different concentrations of MPA and CPZ. **Tables 1** showed that CPZ had a strong inhibitory effect on the viability of ISK and KLE cells; this effect occurred in a time-dependent manner and was following 24, 48, and 72 h, of incubation (**Supplementary Figure 1**). The inhibitory action of CPZ was stronger than that of MPA over the same time periods. To further investigate the anticancer effects of CPZ, we determined IC_50_ values to examine the differential effects between CPZ and MPA. **Table 2** showed that when compared with MPA, CPZ exhibited a much greater inhibitory action on the cell viability of AN3CA cells. CPZ also exhibited stronger anti-tumor effects than MPA in HEC-1-A and HEC-1-B cells. The colony formation assay was also used to investigate the potential role of CPZ in proliferative activity; we observed that CPZ reduced the tendency for proliferation (**Figures 1A, B**). The staining of cells with annexin V/PI further demonstrated that the rates of apoptosis in CPZ-treated EC cells were significantly higher than in cells treated with MPA (**Figures 1C, D**). These findings indicated that the process of apoptosis was activated by CPZ. As shown in **Figures 3E, F**, CPZ treatment led to a reduction in the capability of ISK and KLE cells to migrate and invade when compared with cells that had been treated with MPA. Thus, our results demonstrated that CPZ could exert significant effects on proliferation, migration, and invasion in EC cell lines, and could also exert influence on apoptosis.

**Table 1:** MPA and CPZ were able to inhibit the proliferation of ISK and KLE cells.

Time (h)	ISK			KLE		
	IC_50_ of MPA(μM)	IC_50_ of CPZ (μM)	MPA(IC_50_)/CPZ(IC_50_)	IC_50_ of MPA(μM)	IC_50_ of CPZ (μM)	MPA(IC_50_)/CPZ(IC_50_)
24	>100	33.14±2.28	>3.02	>100	35.62±3.18	>2.81
48	29.30±1.05	22.09±3.27	1.32	40.68±2.23	25.54±1.70	1.59
72	21.66±1.50	22.75±0.89	0.95	34.54±2.85	9.97±1.22	3.46

Results are expressed as the mean of at least three replicates.

**Table 2:** The 72-hour abilities of MPA and CPZ to inhibit proliferation in other EC cell lines.

Cell lines	IC_50_ of MPA (μM)	IC_50_ of CPZ (μM)	MPA(IC_50_)/CPZ(IC_50_)
AN3CA	35.21±1.12	18.77±3.50	1.88
Hec-1-A	59.59±3.41	23.15±1.14	2.57
Hec-1-B	36.72±2.18	17.88±0.64	2.05

Results are expressed as the mean of at least three replicates.

### CPZ Suppressed the Growth of EC Tumors *In Vivo*

**Figure 2:**
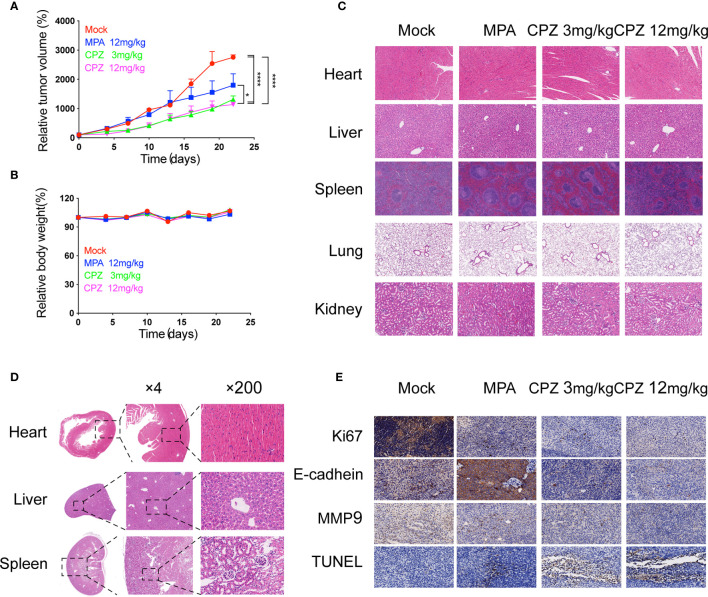
Antitumor efficacy and toxicity assessment of CPZ in ISK xenograft model. **(A)** Tumour size and **(B)** body weight measurements from ISK xenograft mice after CPZ. We set the initial body weight and tumor size to 100%. **(C)** CPZ treatment did not have a toxic effect on any of the five major organs. **(D)** H&E-stained sections of heart, liver, and kidney from a mouse treated with CPZ (12mg/kg) revealed that the tissue and cell architecture were both normal. The area of tissue marked by the dotted square is shown at a higher magnification in the right panel **(E)** Immunohistochemical staining assay. (^*^p < 0.05, ^****^p < 0.0001, two-way analysis of variance with Tukey’s multiple comparisons test). Data expressed as mean ± standard error of the mean.

Next, we investigated whether CPZ could exert similar effects *in vivo*. We established murine xenograft models using ISK cells. CPZ exerted a much stronger inhibitory effect upon tumor growth than MPA when administered at a dose of 12 mg/kg. When the CPZ dose was reduced to 3 mg/kg, there was still a strong suppressive effect against tumor growth (**Figure 2A**). We also found that the body weight changes in our model mice remained within the normal range (**Figure 2B**). We also used immunohistochemical staining of Ki67 and E-cadherin, along with TUNEL staining, to detect cell proliferation, invasion, and apoptosis, respectively. Levels of Ki67 and E-cadherin were markedly reduced, while the number of TUNEL-positive cells had increased (**Figure 2E**).

### CPZ Exhibited a Good Safety Profile as an Anti-Tumor Drug Candidate

The clinical translation of CPZ has been hindered due to the fact that it can induce non-desirable effects at clinically appropriate doses. We attempted to investigate the safety profile of CPZ, by evaluating the toxicity profile of CPZ *in vivo* using repeat intraperitoneal injections in BALB/c mice. The mice were treated with either MPA or CPZ; we then determined a range of hematological data that could act as indicators of injury to the organs or tissues. Hematological data derived from mice treated with either MPA and CPZ were similar to those derived from the Mock group (**Table 3**). At the end of the treatment period, the mice were culled, and a range of tissues and organs were stained with H&E; there was no specific evidence for organ-related toxicities (**Figure 2C**). As a previously widely used antipsychotic drug, CPZ is mainly metabolized in liver and gradually excreted by kidney. It is reported that CPZ may cause hepatic dysfunction as a side effect (22, 23). We found no statistical differences in the levels of any of the liver or kidney parameters tested in the mice treated with CPZ when compared to those of the Mock group (**Table 3**). Furthermore, histological analysis revealed that tissue appeared to be normal with an appropriate cellular architecture (**Figure 2D**). Collectively, our data provided that CPZ exhibited strong suppressive activity against tumors without toxic side effects *in vivo.*

**Table 3:** Hematological data, and liver and kidney function testing derived from BALB/c mice following daily treatment with MPA or CPZ.

Analyte (units)	Mock	MPA (12mg/kg)	CPZ (3mg/kg)	CPZ (12mg/kg)
RBC(× 10^12^/L)	6.80±2.46	7.96±2.23	7.65±1.17	8.79±0.37
HGB(g/L)	105.25±42.00	124.33±38.94	116.60±19.67	133.25±11.81
HCT(%)	35.80±12.70	39.83±12.85	38.60±5.79	46.28±4.04
MCH(pg)	15.35±1.39	15.47±0.65	15.16±0.80	15.13±1.13
PLT(10^9^/L)	729.00±443.13	784.67±471.58	695.00±287.98	880.00±464.65
AST(U/L)	399.49±134.59	305.29±187.27	237.92±51.98	243.45±25.05
ALT(U/L)	79.70±46.23	46.47±14.21	50.69±9.03	66.17±8.40
UREA(mmol/L)	10.49±1.01	12.16±1.02	9.05±1.87	10.46±1.68
CREA(μmol/L)	26.12±2.45	26.83±3.04	22.69±2.68	27.33±1.69

Data shown represent means ± standard deviation. RBC, red blood cells; HGB, hemoglobin; MCH, mean cell height; HCT, hematocrit; PLT, platelets; AST, aspartate aminotransferase; CREA, creatinine; UREA, carbamide; ALT, alanine aminotransferase.

### Concomitant Treatment Induces Apoptosis in ISK and KLE Cells

**Figure 3:**
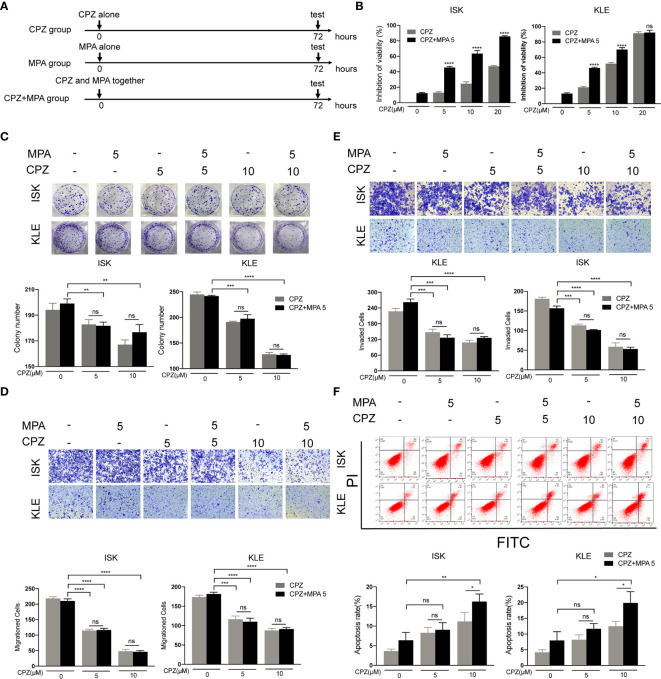
Concomitant treatment induces apoptosis in ISK and KLE cells. **(A)** Schematic representation of the combination treatment on ISK and KLE cells. Cells were divided into three groups: MPA group (MPA alone), CPZ group (CPZ alone) and MPA + CPZ group (CPZ and MPA together). **(B)** ISK and KLE cells were exposed to CPZ or MPA as single drugs or in simultaneous combination for 72 h. **(C)** The colony formation assay was used to evaluate the formation of colonies by ISK and KLE cells when treated with a combination of CPZ and MPA. **(D, E)** Migratory and invasive ability was assessed in ISK and KLE cells subjected to different treatment regimens. **(F)** We used flow cytometry to determine the rate of apoptosis in ISK and KLE. Results are presented as mean ± standard error of the mean and error bars represent the SD of three independent experiments. ns, not significant; ^*^p < 0.05; ^**^p < 0.01; ^***^p < 0.001; ^****^p < 0.0001.

Since MPA and CPZ both possess antitumor effect, we wondered whether combination treatment with MPA and CPZ exhibited synergistic effect. Firstly, we assessed the effect of concomitant treatment with the two drugs (MPA and CPZ administrated together) (**Figure 3A**). CCK-8 assays showed that concomitant treatment decreased the viability of ISK and KLE cell lines compared to MPA or CPZ treatment alone (**Figure 3B**). The inhibition action of 5 μM CPZ plus 5 μM MPA is not only close to 10 μM CPZ alone in KLE cells but also stronger than 10 μM CPZ alone in ISK cells. Interestingly, colony formation assays and Transwell assays proved that the inhibition of proliferation and migration/invasion in concomitant treatment was stronger than MPA treatment alone, but similar to CPZ treatment alone in ISK and KLE cells (**Figures 3C–E**). Though high dose of CPZ co-treatment with MPA could enhance apoptotic cell rates in both cells, low dose of CPZ showed no difference (**Figure 3F**). These data demonstrated that concomitant treatment only performed a limited synergistic effect on apoptosis in ISK and KLE cells. Considering that combination treatment includes concomitant treatment and sequential treatment (24, 25), we judged whether another treatment is better than this one in the following experiments.

### Sequential Treatment Sensitizes KLE Cells to MPA

**Figure 4:**
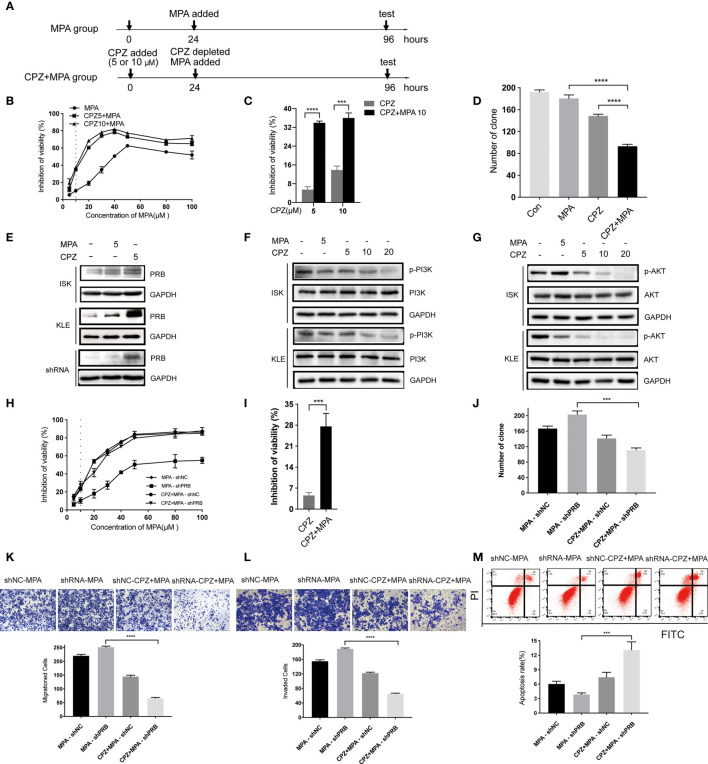
CPZ increases progesterone-resistant cells sensitivity to MPA by upregulating PRB. **(A)** Schematic representation of the sequential treatment on KLE and shRNA cells. Cells were divided into two groups: MPA group (cultured for 24h, MPA added, cultured for 72h, then test) and CPZ + MPA group (CPZ added, cultured for 24h, CPZ depleted, MPA added, cultured for 72h, then test). **(B, C)** After 24 h exposure to CPZ of 5 μM and 10 μM, cells were administered with various concentrations of MPA; cells were then analyzed by a Coulter Counter 72 h later. **(D)** Pre-treatment with CPZ before MPA treatment decreases the colony formation of KLE cells as compared to MPA or CPZ treatment alone. Cells were pre-treated with CPZ for 24 h before MPA treatment for 72 h. **(E)** CPZ upregulated the protein level of PRB in KLE and shRNA cells treated with 5 μM CPZ for 24h. **(F) **Effect of CPZ on PI3K activity was assayed by immunoblotting for total PI3K and phosphorylated PI3K ^Tyr467/Tyr199^. **(G)** Expression levels of AKT and p-AKT^ser473^ in ISK and KLE cell lines when treated with CPZ. **(H, I)** Pre-treatment with CPZ for 24h, shRNA cells were incubated with MPA at various concentrations for 72 h. **(J)** CPZ decreased MPA-induced colony formation in transfected ISK cells. **(K, L)** Migration and invasion were observed in transfected ISK cells. **(M)** Pre-treatment with CPZ, the apoptosis rate of transfected ISK cells in response to MPA. Results are presented as mean ± standard error of the mean and error bars represent the SD of three independent experiments. ^***^p < 0.001; ^****^p < 0.0001.

Based on the above results, we tested the effect of sequential treatment on KLE cells (24 hours of CPZ, followed by 72 hours of MPA; 48 hours of CPZ, followed by 48 hours of MPA) (**Figure 4A** and **Supplementary Figure 2A**). Both low dose (5 μM) and high dose (10 μM) CPZ could obviously enhance the cytotoxic effect of MPA on KLE cell lines after pre-treatment with CPZ for 24h (with 2.5 times enhancement at both doses) (**Figure 4B**). Furthermore, a nearly 6-fold increase of inhibition rate was recorded in sequential treatment than in low dose CPZ treatment alone (**Figure 4C**). Pre-treatment with CPZ could also significantly suppress the colony formation of KLE cells compared to single drug treatment (**Figure 4D**). There is no difference between pre-treatment for 24 hours groups and pre-treatment for 48 hours groups, when it comes to CPZ treatment alone (**Supplementary Figures 2B–D**). These data suggested that sequential treatment with CPZ and MPA showed synergistic effect in progesterone-resistant cells. This provided a new strategy of sequential treatment, which shortened duration and usage of CPZ to reduce side effect.

### CPZ Increases Progesterone-Resistant Cells Sensitivity to MPA by Upregulating PRB

MPA inhibits the growth of normal and cancerous endometria mainly *via* PRB (26). To investigate the mechanism of CPZ in sensitizing EC cells to MPA, we tested PRB level after CPZ treatment for 24 hours. Compared with MPA group, PRB expression in CPZ group was upregulated parentally in KLE cells, while it was increased slightly in ISK cells (**Figure 4E**). These results suggested that the sensitivity of KLE cells to MPA was associated with PRB expression. Then, PRB was knocked down in ISK cells to verify if pre-treatment with CPZ enhanced progesterone-resistant cells sensitivity to MPA *via* upregulating PRB (**Supplementary Figure 2E**). Compared with MPA-shRNA group, the sensitivity of CPZ+MPA-shRNA group to MPA was significantly decreased (**Figure 4H**). Behavioral experiments including CCK-8 assays, colony formation assays, Transwell assays and flow cytometry presented that pre-treatment with CPZ could restore progesterone-resistant cells sensitivity to MPA(**Figures 4H–M**). At the same time, PRB expression increased remarkedly after CPZ treatment in shRNA cells (**Figure 4E**). Together, these data indicated that CPZ increased progesterone-resistant cells sensitivity to MPA by upregulating PRB.

Recent studies suggested that the inhibition of hyperactive AKT signaling can increase PRB-dependent transcription, which plays a putative role in the development of resistance to progestin in EC (27, 28). Thus, the activity of PI3K/AKT in both EC cells was detected to explore the mechanism of PRB up-regulation by CPZ pre-treatment. Following CPZ treatment, phosphorylation of PI3K^Tyr467/Tyr199^ and AKT^Ser473^ was significantly inhibited in a dose-dependent manner (**Figures 4F, G**). These data indicated that the CPZ upregulated PRB by inhibiting the PI3K/AKT pathway and then sensitized progesterone-resistant cells to MPA.

## Discussion

Repurposing CPZ exhibited much advantage in various cancers including glioblastoma, lung cancer, colon cancer and breast cancer (18–20). There were no relevant studies of CPZ in endometrial cancer at present. In this study, we explored the anti-endometrial cancer effects of CPZ. CPZ exerted inhibitory effects on EC both *in vitro* and *in vivo* indicating that CPZ showed good application prospect in EC. However, previous research has shown that antipsychotic drugs had extensive cardiotoxicity (29, 30) and were extensively metabolized by hepatic oxidation (31, 32). Histological analysis and biochemical analyses failed to identify evidence of obvious tissue toxicity after CPZ treatment. The possible explanation is that there is toxicity at higher dose but no toxicity at this test dose.

CPZ was well-known as an antagonist of dopamine receptor D2(DRD2) (18). It was reported that dopamine acting through DRD2, down regulated insulin-like growth factor-1 (IGF-1) and AKT phosphorylation, thereby inhibited gastric cancer proliferation (33). There was a significant increase in the expression of IGF-1R in EC compared with normal endometrium (34). IGF-I promoted the growth of EC cells *in vitro* (35). High expression of IGF-1R, by binding with IGF, could activate PI3K/AKT signaling pathway to promote cell growth, proliferation and anti-apoptosis (35–37). Possible mechanism is that CPZ, by acting through DRD2, inhibits IGF-I-induced EC development through down-regulation of IGF-IR and PI3K/AKT phosphorylation.

Combination treatment including concomitant treatment and sequential treatment was proceeded to explore the synergistic effect of CPZ and MPA. Sequential treatment showed favorable synergistic effect in progestin-resistant cells compared with concomitant treatment; Pre-treatment with low dose CPZ (5 μM) could upregulate PRB expression effectively. Together, sequential treatment with CPZ and MPA could reduce side effects by shortening the duration and reducing the dosage. It was reported that ISK cell line with long-term progestin treatment could downregulate PR expression and turned to a progestin-resistant ISK cell line (38). Since CPZ could increase PRB expression in shRNA and KLE cells, sequential treatment with CPZ and MPA could be an ideal drug for patients whose PR expression was quite low or changed to resistant from sensitive.

In accordance with the current rules, by the Food and Drug Administration (FDA), the new molecular drug approval spans over 10 years and the overall cost reaches $868M USD (39). Given that CPZ has come into clinical use since the 1950s, all the data concerning its pharmacology and toxicology as a single drug are well established. Such knowledge enables us to bypass the Phase I clinical investigation and proceed directly to a Phase II clinical investigation (18, 36), which could save time and cost to a large extent. In conclusion, CPZ may represent a suitable therapeutic option for patients with EC.

## Data Availability Statement

The original contributions presented in the study are included in the article/**Supplementary Material**. Further inquiries can be directed to the corresponding authors.

## Ethics Statement

The animal study was reviewed and approved by Shanghai Jiao Tong University (GKLW) 2018-40.

## Author Contributions

Conceptualization: YC. Methodology: YC, TH, XG, HW, and LL. Formal analysis and investigation: YC and LY. Writing - original draft preparation: YC. Writing - review and editing: LY and XS. Funding acquisition: YW. Resources: YC and JL. Supervision: XS and FM. All authors contributed to the article and approved the submitted version.

## Funding

This study was funded by Shanghai Municipal Key Clinical Specialty (No. shslczdzk06302), National Natural Science Foundation of China (No. 81172477, 81402135,22077033), the Project of the Science and Technology Commission of Shanghai Municipality (No. 17441907400) and Shanghai Jiao Tong University Medicine-Engineering Fund (No. YG2017MS41), National Natural Science Foundation of China (No. 20ZR1414800).

## Conflict of Interest

The authors declare that the research was conducted in the absence of any commercial or financial relationships that could be construed as a potential conflict of interest.

## Supplementary Material

Supplementary Table 1 Proliferation inhibition of 20 tricyclic antipsychotic drugs in ISK cell lines.

Supplementary Figure 1 The proliferation curve of ISK and KLE cells. ISK and KLE cells were treated with different concentration of MPA and CPZ for 24,48 and 72h. Data are shown as the mean ± SEM; n = 3.

Supplementary Figure 2 CPZ sensitizes KLE cells to MPA by upregulating PRB. (A)Schematic representation of the sequential treatment on KLE cells. (B-C) Pre-treatment with 5 μM and 10 μM CPZ for 48h, KLE cells then were incubated with various concentrations of MPA for 48h. (D) CPZ upregulated the protein level of PRB in KLE cells treated with 5 μM CPZ for 48h. (E) The transfected efficiency was measured by Western Blot. Results are presented as mean ± standard error of the mean and error bars represent the SD of three independent experiments. **p<0.01.
