# Supplementary material for: Chlorpromazine Sensitizes Progestin-Resistant Endometrial Cancer Cells to MPA by Upregulating PRB
Source: Front Oncol. 2021 Apr 16;11:665832. doi: 10.3389/fonc.2021.665832 (PMC8087176; doi:10.3389/fonc.2021.665832)
Supplement: Supplementary Table 1 — Proliferation inhibition of 20 tricyclic antipsychotic drugs in ISK cell lines. [file DataSheet_1.pdf]

Drug	Inhibition @40 μM (48h, %)
Tianeptine	6.81
Olanzapine	3.14
Doxepin hydrochloride	7.62
Clozapine	13.09
Chlorpromazine hydrochloride	78.92
Quetiapine fumarate	37.47
Remeron	19.31
Trifluoperazine hydrochloride	86.00
Perphenazine	78.94
Amoxapine	16.12
Melitracen hydrochloride	0.33
Imipramine hydrochloride	11.90
Nortriptyline hydrochloride	46.89
Trimipramine maleate	35.92
Maprotiline hydrochloride	45.15
Amitriptyline hydrochloride	28.58
Thioridazine hydrochloride	87.76
Mianserin hydrochloride	7.60
Clomipramine hydrochloride	3.21
Chlorprothixene	47.18
