# Supplementary figures and images for: Chlorpromazine Sensitizes Progestin-Resistant Endometrial Cancer Cells to MPA by Upregulating PRB
Source: Front Oncol. 2021 Apr 16;11:665832. doi: 10.3389/fonc.2021.665832 (PMC8087176; doi:10.3389/fonc.2021.665832)

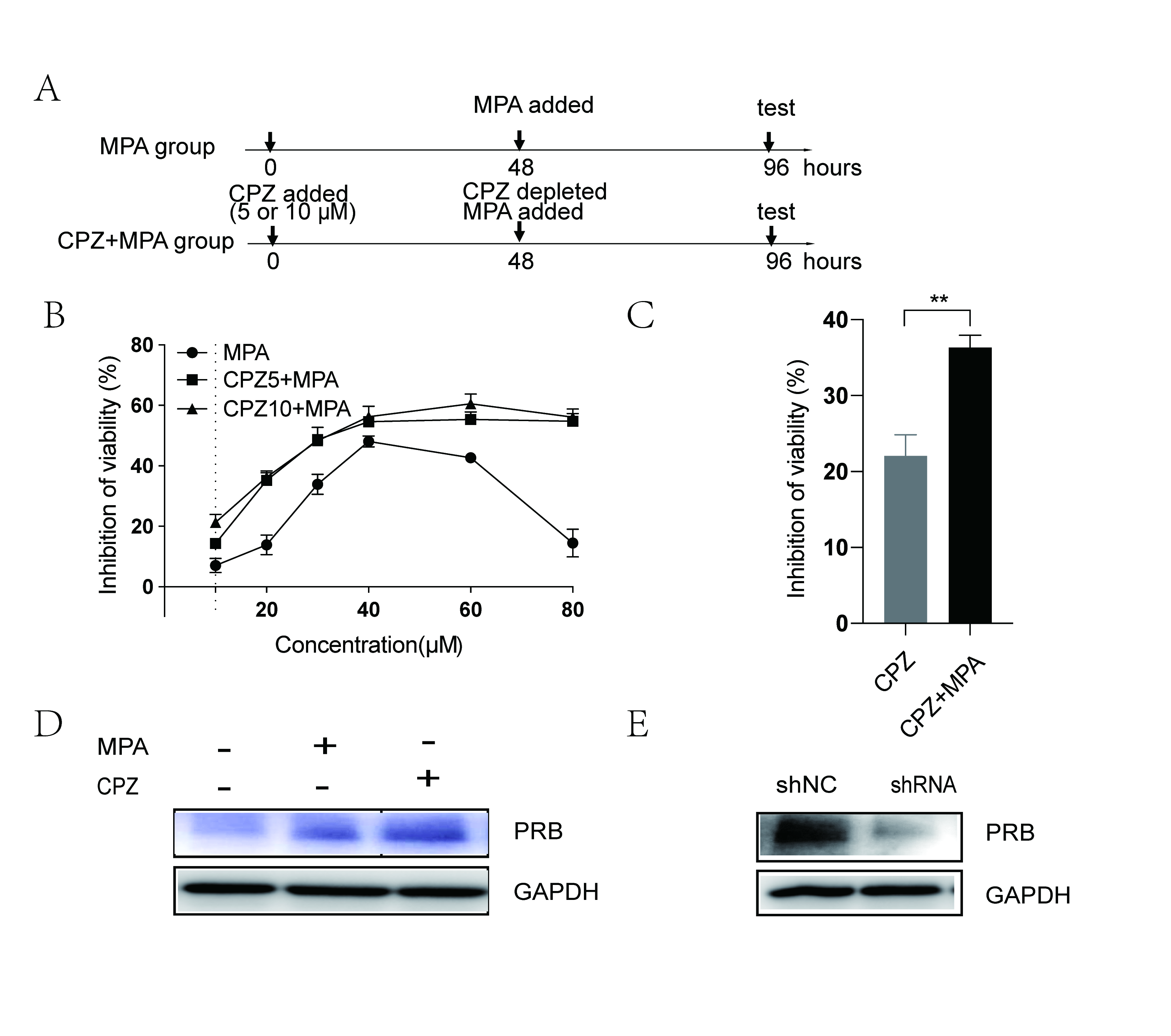

Supplement: Supplementary Figure 1 — The proliferation curve of ISK and KLE cells. ISK and KLE cells were treated with different concentration of MPA and CPZ for 24,48 and 72h. Data are shown as the mean ± SEM; n = 3. [file Image_1.tif]

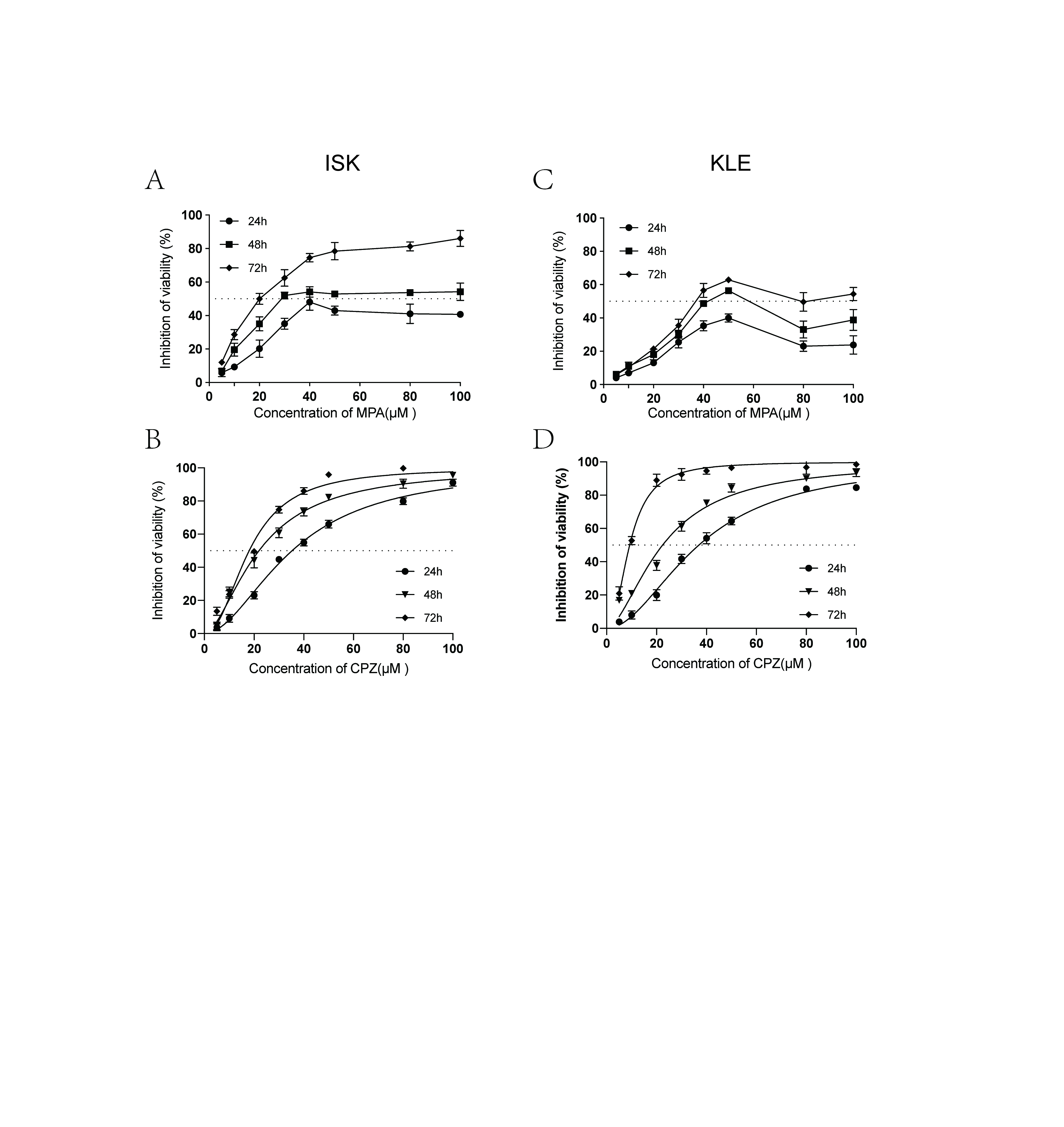

Supplement: Supplementary Figure 2 — CPZ sensitizes KLE cells to MPA by upregulating PRB. (A)Schematic representation of the sequential treatment on KLE cells. (B-C) Pre-treatment with 5 μM and 10 μM CPZ for 48h, KLE cells then were incubated with various concentrations of MPA for 48h. (D) CPZ upregulated the protein level of PRB in KLE cells treated with 5 μM CPZ for 48h. (E) The transfected efficiency was measured by Western Blot. Results are presented as mean ± standard error of the mean and error bars represent the SD of three independent experiments. **p<0.01. [file Image_2.tif]
